# Pan-precancer and cancer DNA methylation profiles revealed significant tissue specificity of interrupted biological processes in tumorigenesis

**DOI:** 10.1080/15592294.2023.2231222

**Published:** 2023-07-02

**Authors:** Feifan Zhang, Xin Zhang, Haikun Zhang, Dongdong Lin, Hailang Fan, Shicheng Guo, Fang An, Yaqian Zhao, Jun Li, Steven J. Schrodi, Dake Zhang

**Affiliations:** a Key Laboratory of Biomechanics and Mechanobiology, Ministry of Education, Beijing Advanced Innovation Center for Biomedical Engineering, School of Engineering Medicine, Beihang University, Beijing, China; b Department of Urology, Beijing Chaoyang Hospital, Capital Medical University, Beijing, China; c Department of Medical Genetics, University of Wisconsin-Madison, Madison, WI, USA; d Department of Obstetrics and Gynecology, Peking University People’s Hospital, Beijing, China; e Department of Gastroenterology, Peking University Third Hospital, Beijing, China; f Computation and Informatics in Biology and Medicine, University of Wisconsin-Madison, Madison, WI, USA

**Keywords:** Precancer, DNA methylation, tumorigenesis

## Abstract

DNA methylation (DNAme) alterations are known to initiate from the precancerous stage of tumorigenesis. Herein, we investigated the global and local patterns of DNAme perturbations in tumorigenesis by analysing the genome-wide DNAme profiles of the cervix, colorectum, stomach, prostate, and liver at precancerous and cancer stages. We observed global hypomethylation in tissues of both two stages, except for the cervix, whose global DNAme level in normal tissue was lower than that of the other four tumour types. For alterations shared by both stages, there were common hyper-methylation (sHyperMethyl) and hypo-methylation (sHypoMethyl) changes, of which the latter type was more frequently identified in all tissues. Biological pathways interrupted by sHyperMethyl and sHypoMethyl alterations demonstrated significant tissue specificity. DNAme bidirectional chaos indicated by the enrichment of both sHyperMethyl and sHypoMethyl changes in the same pathway was observed in most tissues and was a common phenomenon, particularly in liver lesions. Moreover, for the same enriched pathways, different tissues may be affected by distinct DNAme types. For the PI3K−Akt signalling pathway, sHyperMethyl enrichment was observed in the prostate dataset, but sHypoMethyl enrichment was observed in the colorectum and liver datasets. Nevertheless, they did not show an increased possibility in survival prediction of patients in comparison with other DNAme types. Additionally, our study demonstrated that gene-body DNAme changes of tumour suppressor genes and oncogenes may persist from precancerous lesions to the tumour. Overall, we demonstrate the tissue specificity and commonality of cross-stage alterations in DNA methylation profiles in multi-tissue tumorigenesis.

## Introduction

In recent years, DNA methylation (DNAme) alterations, as hallmarks of tumorigenesis, have been widely used as promising biomarkers for tumour diagnosis and detection [1,2]. In cells, the DNA methyltransferases catalyse the transfer of a methyl group from S-adenyl methionine to the fifth carbon of a cytosine residue to produce 5-methylcytosine [3]. DNAme mostly occurs on a cytosine that is located at 5’ of a guanine nucleotide, known as CpG (cytosine-phosphate-guanine) site [4]. Although DNAme is the most stable type of epigenetic modification regulating the transcriptional plasticity of mammalian genomes [5], it is also dynamic and can be affected by various factors such as abnormal expression of DNA methyltransferases in tumour cells [6,7]. Global DNA hypomethylation accompanied by focal hypermethylation is a well-recognized feature in the tumour genome [8].

Methylation of CpG sites in gene promoters can hinder transcription factor binding, or recruit repressive methyl-binding proteins, thereby silencing gene expression. Altered DNAme in gene promoters has long been found to contribute to tumorigenesis by regulating the activities of tumour suppressor genes (TSG) [9]. TSG silencing due to promoter methylation has attracted much attention [10]. The hypomethylation of oncogenes is also a well-established mechanism for the abnormal activation of tumour cells [11]. In addition, DNAme can silence the transcription of transposable elements to maintain genome stability [12]. The complexity of the transcriptional regulatory role of DNAme has been revealed by genome-wide analysis. High gene-body methylation and low promoter methylation are associated with the high expression of the corresponding genes [13]. In addition to gene expression regulation, DNAme may assist the spliceosome in exon usage, thus affecting alternative splicing [14]. It has been proposed that intragenic DNAme may initiate the formation of a chromatin structure that impedes transcription elongation [15]. Other studies have further highlighted the role of DNAme in preventing abnormal usage of either intragenic transcription sites [16], or intragenic promoters [17]. Recent studies have illustrated that the methylation of the untranslated region (UTR) is associated with enhanced gene expression [18,19]. Although the regulatory roles of DNAme have not been fully understood, it is believed that abnormal DNAme alterations can contribute to tumorigenesis and the Epigenome-Wide Association Study of the tissues also indicates that epigenetic changes are associated with the risk of tumour development [20,21].

Since there is tissue-specificity of DNAme patterns in maintaining transcriptional programmes and regulating gene expression, tissue-specific changes are expected in cancers [22]. Tissue-specific DNAme markers carried by circulating tumour DNA in the blood provide a solution for determining the origin of tumours in liquid biopsy [23–25]. It is well-known that in addition to DNAme changes, there are extensive genomic alterations in tumours, such as point mutations and structural variants. Moreover, DNAme is known to increase mutation probability at cytosine residues [26,27], while the mutagenesis in the DNA double-strand break repair may also alter the methylation level of the repaired DNA [28]. Currently, the methylation alterations during carcinogenesis seem to occur in genes or genomic regions non-specifically, and tumour mutations also occur randomly. Therefore, the key changes underlying the reprogramming of normal somatic cells into tumour cells are confounded by the accumulated stochastic genomic variations, leading to so-called ‘chaos’ in the tumour epigenome or DNAme profiles [29]. The numerous alterations may also contribute to the considerable individual variations in tumour development, as well as heterogeneity across cell populations within tumours.

However, whether some DNAme alterations persist from precancerous lesions to tumours despite increased genomic instability is unclear. Previously, we profiled the methylation changes in low-grade adenoma (LGA) and high-grade adenoma (HGA) precancerous lesions of colorectal cancer, and found common DNA alterations in both precancerous lesions and tumours, which may become promising biomarkers for early detection of colorectal cancer [30]. Here, we further combined our colorectal data with public cervical, gastric, prostatic, and hepatic data from precancerous lesions with relatively large sample sizes, and characterized the common and specific methylation changes in precancerous lesions and tumours from distinct tissue origins.

## Materials and methods

### Data collection

The public datasets containing the Illumina DNAme microarray (450K/850K) from 5 body sites were downloaded, including the cervix, colon, stomach, prostate, and liver tissues. All the datasets of tissue contained samples of normal tissue, precancerous lesions, and tumours. The corresponding clinical information of patients was also collected. 1) there were 356 samples in the cervical data, including 23 normal samples and 17 cervical epithelial neoplasia (CIN) samples from the GSE135446 dataset, and 9 cervical cancer samples from the GSE135446 dataset, and 307 cervical cancer samples from TCGA; 2) colorectal data contained 232 samples, including 20 normal samples from GSE68060, 25 normal samples from TCGA, 40 precancerous samples (18 low- and 22 high-grade adenomas) from our previous study GSE139404, and 147 colorectal cancer samples from TCGA; 3) gastric data included 516 samples, including 42 normal samples from the GSE99553 dataset, 2 normal samples from TCGA, 76 precancerous samples (intestinal metaplasia) from GSE103186, and 396 gastric cancer samples from TCGA; 4) prostatic data contained 570 samples, including 50 normal samples from TCGA, 18 precancerous samples (cancer-related fibroblasts) from GSE115413, and 502 tumour samples from TCGA; 5) hepatic data included 560 samples, including 50 normal samples from TCGA, 130 cirrhotic tissue samples from GSE157973, and 380 tumour samples from TCGA.

We collected low and high-grade adenomas (LGA and HGA) samples (GSE139404) from patients admitted to the Department of Gastroenterology of Peking University Third Hospital. All patients were treatment-naive before admission and underwent endoscopic treatment for colorectal adenoma. The study protocol conformed to the ethical guidelines of the 1975 Declaration of Helsinki and was approved by the Ethics Committee of Peking University Third Hospital (IRB number 206H005). Written informed consent was obtained from all participants.

### Pre-processing of microarray data

DNAme data were analysed by the R package ChAMP (version 2.21.1) [31], and the low-quality or no-signal probes were excluded using the champ.filter function (with default parameters). The probes with over 20% missing values were also removed, and the remaining missing values were imputed with the champ.impute function. Overlapping qualified probes in both 450K and EPIC data were retained for further analysis.

After quality control, 1) gastric data contained 44,219 and 42,596 sites before and after filtering, respectively; 2) colon data included 69,758 and 69,758 sites before and after filtering, respectively; 3) prostate data contained 369,038 sites before filtering and 336,722 sites after filtering; 4) there were 454,213 sites for cervical data before filtering and 385,947 sites after filtering; 6) for hepatic data, there were 394,788 sites before filtering and 360,117 sites after filtering. The final beta matrix was normalized using the champ.norm function (with default parameters). The average β values for all probes after quality control were calculated to indicate the average DNAme (aDNAme) level for each sample.

### Identification and genomic annotation of differentially methylated probes (DMPs)

Using normal samples as reference, we assessed the potential DMPs in the group for comparison using the champ.DMP function. Hyper DMPs were defined as logFold-Change >0.1 and adjusted *P* < 0.05 after Benjamini – Hochberg correction, and hypo DMPs had logFold-Change < −0.1 and adjusted *P* < 0.05. Genomic annotation of DMPs (TSS200, TSS1500, 1stExon, 5´UTR, Gene body, 3´UTR, CGI, Shelf, Shore, and Open Sea) was derived from the ChAMP package.

### Enrichment analysis of DMPs in autosomal chromosomes, genomic features, and gene sets

We estimated the probability of N probes on a selected certain chromosome when sampling D probes in total from a probe set with the size of M, according to the corresponding frequency in the simulation repeated 10,000 times. There was an autosome extracted probe number distribution for each chromosome, and the rate for N selected probes approached the probability when simulation times increased towards infinity. In brief, for hyper DMPs, 2,202 probes were randomly sampled from 732,724 total probes in the array 10,000 times, and for hypo DMPs, 1,864 probes were sampled at each time.

Genomic feature enrichment of eight types of DMPs was performed with the Genomic Association Tester (GAT). The features included exon, intron, intergenic, promoter, 3’ UTR, and 5’ UTR, and the default parameters were used to calculate the significance [32]. For Gene Ontology(GO) and Kyoto Encyclopedia of Genes and Genomes (KEGG) enrichment of DMP-related genes, the R package clusterProfiler (version 4.2.2) with default parameters was used [33].

### Annotation and expression analysis of oncogenes and TSGs (Onco/TSGs)

The Onco/TSGs were annotated according to the census file retrieved from https://cancer.sanger.ac.uk/census (Census_allSun Jun 19 16_24_02 2022) [34]. Sites were identified if they were located at gene body, UTRs and promoter of Onco/TSGs. Their expression levels in the TCGA database were analysed and visualized with the GEPIA2 (http://gepia2.cancer-pku.cn/) [35].

### Survival analysis

To evaluate the contribution of eight types of DMPs to the tumour prognosis, clinical data of patients in TCGA database were extracted for overall survival (OS) analysis using the R package survival (version 3.3–1, https://CRAN.R-project.org/package=survival). The TCGA dataset was divided into the training dataset and the validation dataset at the ratio of 7:3. The univariate Cox proportional hazard analysis was first conducted in the training dataset to identify significant methylation markers (*p* < 0.05) associated with OS. Then, the multivariate Cox regression analysis was performed on the significant variables. The groups with long- and short-term OS were divided. The Overall survival (OS) and Disease-free survival (DFS) curves were plotted using the Kaplan – Meier method and analysed with the log-rank test.

## Results

### Fluctuation of global DNAme levels between precancerous lesions and tumour tissues

**Figure 1.**
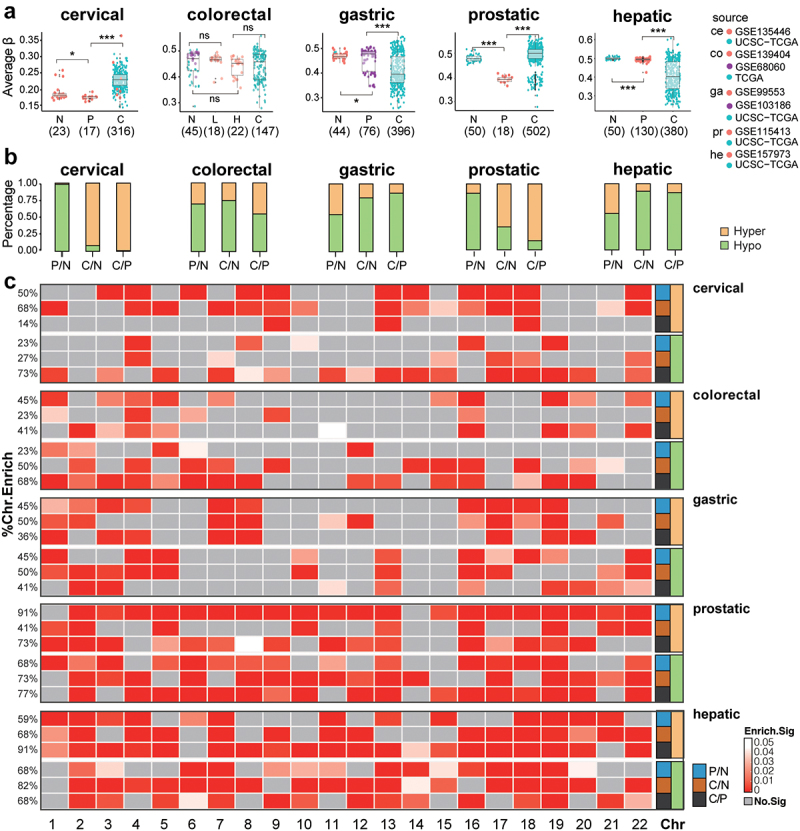
Overall DNA methylation (DNAme) changes in precancerous and tumour tissues for all five tissue types. a. the distribution of average DNAme levels (aDname) in normal tissues (N), precancerous lesions (P), and cancer samples (C) of five tissue types. The dot colours indicate the data source. ce, cervical; co, colorectal; ga, gastric; pr, prostatic; he, hepatic. * represents for *P* ≤ 0.05, ** represents for *P* ≤ 0.01, *** represents for *P* ≤ 0.001. b. Percentages of hypomethylation (hypo) and hypermethylation DMPs (differentially methylated probes). c. Chromosome enrichment for DMPs of three comparisons.

In the colorectal samples that were collected in our previous study, the global DNAme levels decreased in HGA than in LGA (Figure 1a). However, the DNAme levels evaluated by Illumina DNAme microarray (aDNAme, **Method**) in colon cancer samples from TCGA were not lower than those of our LGA and HGA samples, a large proportion of which were even higher than normal samples. To explore DNAme alterations in precancerous lesions, we collected genome-wide methylation microarray data from three stages normal tissues, precancerous lesions, and cancer tissues of the cervix, colorectum, stomach, prostate, and liver. Principal component analysis (PCA) of aDNAme revealed global tissue-specific clustering in normal tissues and precancerous lesions, with colorectum and stomach exhibiting close clustering. In contrast, different groups of cancer tissues clustered together and displayed dispersion (**Supplementary Figure S1**). Changing trends for aDNAme were different in tumorigenesis among these tissues (Figure 1a). In the colon and prostate, aDNAme decreased in the precancerous lesions and increased in the tumour tissues. In contrast, in hepatic and gastric samples, the aDNAme level was not obviously changed in the precancerous lesions, but decreased in the tumour tissues. Meanwhile, the overall methylation alteration in the precancerous lesions was mild as evidenced by similar methylation levels between precancerous and normal samples in all tissues, except for prostatic samples, whose precancerous lesions had significantly decreased aDNAme levels (Figure 1a).

Genome-wide DNAme levels also exhibited tissue differences. Normal and precancerous samples from the colorectum, stomach, prostate, and liver had relatively consistent aDNAme levels, with the average β value varying in the range of 0.40–0.50 (Figure 1a). However, the normal, precancerous, and tumour samples of the cervix all had very low aDNAme levels (average β, normal: 0.18; precancer: 0.18; tumour: 0.23, Figure 1a). In particular, within-group differences varied in each stage. In general, cancer samples showed substantial individual aDNAme differences, but precancerous and normal tissues had limited variations in aDNAme across different samples. Among five datasets, the normal tissues and hepatic precancerous showed minimal intra-group variations in aDNAme (CV, 0.013 and 0.016, respectively). Meanwhile, the normal tissues and precancerous lesions of the colorectum and stomach showed large intra-group differences (CV of normal tissues, LGA and HGA samples were 0.013, 0.017 and 0.03 in colorectum; CV of the normal tissues and precancerous lesions were 0.02 and 0.1 in stomach).

Epigenetic reprogramming in tumorigenesis may not be the same in distinct tissues. In comparison with normal tissues, hypo DMPs of cancer in the colorectal, gastric and hepatic cancer tissues (Figure 1b, C/N) accounted for the majority of all DMPs (over 70%) (74.3%, 79.4%, and 88.7% respectively), while cervical and prostatic cancers had significantly more hyper DMPs than hypo DMPs (91.6% for cervical cancer vs. 65.2% for prostatic cancer, Figure 1b). Generally, normal and precancerous lesions (P/N) had similar aDNAme levels, which indicates mild changes in precancerous methylation profiles.

All autosomal chromosomes had significant enrichment of DMPs in certain stages of tumorigenesis, showing as the genome-wide extensive DNAme changes (*P* < 0.001, Figure 1c). Nevertheless, methylation changes seemed to be more enriched in chromosome 19, which had all types of DMPs enriched in all stages of tumorigenesis in most tissues. Although chromosome 1 was the largest chromosome, its DMP enrichment level among lesions in different stages was relatively low. Meanwhile, DMP enrichment in chromosome 14 was the lowest for all these five tissues. For functional evaluation, similar percentages of DMPs in either all CpG positions (1 ~ 2%, each, **Supplementary Figure S2A**) or all genomic features (~1%, each, **Supplementary Figure S2B**) were constantly observed in all stages of tumorigenesis in all five types of tissues.

### Eight types of DMPs in precancerous lesions and tumours highlight the tissue-specific DNAme alterations

**Figure 2.**
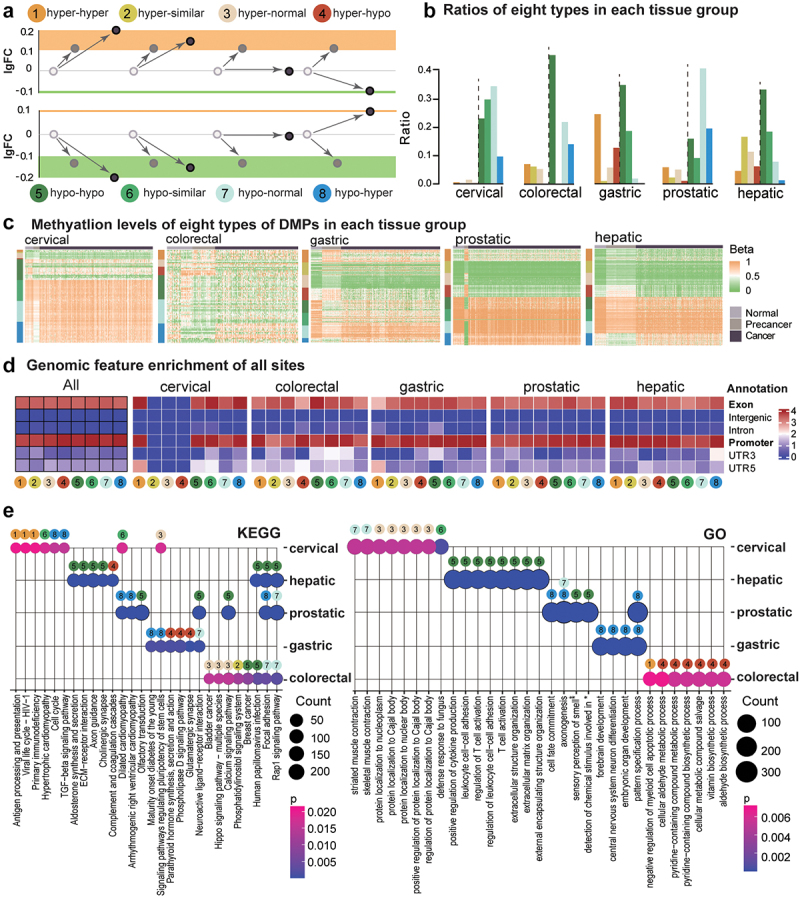
Eight types of methylation alterations across precancerous and tumour stages in all datasets of five tissue origins. a. DNAme fold changes for all eight types. b. Percentages of each type in each tissue origin. c. DNA methylation for DMPs in each sample. Genomic feature enrichment (d) and KEGG and GO enrichment (e) for eight types of DMPs after annotation.

We defined eight types of methylation alterations in precancerous lesions and tumours (Figure 2a): 1) hyper-hyper methylation alterations: the sites were hypermethylated in both precancerous lesions and tumours in comparison to normal tissues, and the methylation level in tumours was even higher, with Δβ _tumour/pre-cancer_ >0.1; 2) hyper-similar methylation alterations: the sites were hypermethylated at a similar level in both precancerous lesions and tumours; 3) hyper-normal methylation alterations: there were hyper DMPs in precancerous lesions and normal methylation levels in tumours; 4) hyper-hypo methylation alterations: there were hyper DMPs in precancerous lesions and hypo DMPs in tumours; 5) hypo-hypo methylation alterations, which used the same standard as described for the previous four hyper-types; 6) hypo-similar methylation alterations; 7) hypo-normal methylation alterations; and, 8) hypo-hyper methylation alterations. Types 1, 2, 5, and 6 indicated the constant or continued changes from the precancerous lesions to the tumours; types 3 and 7 showed methylation changes only observed in the precancerous lesions but not in the tumours; types 4 and 8 showed the opposite changes in the precancerous lesions and tumours. In all, 140,340 sites were found in these five datasets. The methylation changes in precancerous lesions were similar, while heterogeneity was obvious in tumour samples (Figure 2c).

The composition of the 8 types varied somewhat across the five types of tissues, and the major types were the hypo DMPs (55.7%~ 97.4%, Figure 2b). In particular, the hypo-hypo type had the highest percentage in colorectal, gastric, and hepatic tissues from the digestive tract. Interestingly, only the gastric tissue among them also showed a larger proportion of hyper-hyper DMPs (24.7%), and high-methylation epigenotype is a known feature of gastric cancer [36]. In addition, the number of hypo-normal type DMPs was the largest in the prostate (9.2%), which suggests the significant heterogeneity of DNAme alterations during prostatic tumorigenesis. For genomic features, almost all 8 types showed more enrichment in exon and promoter regions in all these tissues (Figure 2d). Although most cervical DMPs had increased methylation levels in precancer and cancer stages, the commonly altered loci shared by two stages were featured by hypomethylation changes. It may require further validation considering the relatively small sample size and the limited amount of DMPs in the cervical dataset.

For the genes annotated for the top 1000 sites (ranking according to *p* values of Precancerous/Normal difference) in each type of tissue, their top 8 most valuable enriched KEGG pathways and GO terms (ranking according to enrichment *p* values, Pearson test) demonstrated substantial tissue specificity (Figure 2e). The cervical DNAme changes, which carried constant hyper DMPs in both precancerous lesions and tumour tissues, were enriched in infection-related KEGG pathways, including Antigen processing and presentation (p = 2.15e-02, Pearson test), Viral life cycle-HIV −1 (p = 2.15e-02, Pearson test), and Primary immunodeficiency (p = 2.15e-02, Pearson test). Meanwhile, in the cervical precancerous with rich hypermethylation changes that were not observed in tumour tissues (Type 3, hyper-normal), the enriched pathways were related to the Cajal body. Hepatic DNAme alterations were dominantly hypo-hypo (Type 5) changes in both precancerous and cancer samples. Their enriched KEGG pathways included Aldosterone synthesis and secretion (p = 1.6e-07, Pearson test), ECM−receptor interaction (p = 4.89e-08, Pearson test), Axon guidance (p = 3.96e-08, Pearson test), and Cholinergic synapse (p = 9.05e-09, Pearson test). Meanwhile, their GO enrichment highlighted the importance of T-cell activation. In the prostatic lesions, hypomethylations were enriched in the KEGG pathway of Olfactory transduction in both prostatic precancerous and cancer tissues (Type 5, hypo-hypo), and GO analysis also showed that Type 5 methylation was enriched in sensory perception of smell (p = 4.67e-39, Pearson test). Nevertheless, these methylation changes in gastric tissues did not show consistent changes in the two stages (Types 4, 7, and 8). Besides, the most commonly affected KEGG pathways were Focal adhesion and Rap1 signalling pathways in hepatic, prostatic, and colorectal datasets.

### Tissue-specific biological processes interrupted by sHypermethyl and sHypomethyl changes in tumorigenesis

**Figure 3.**
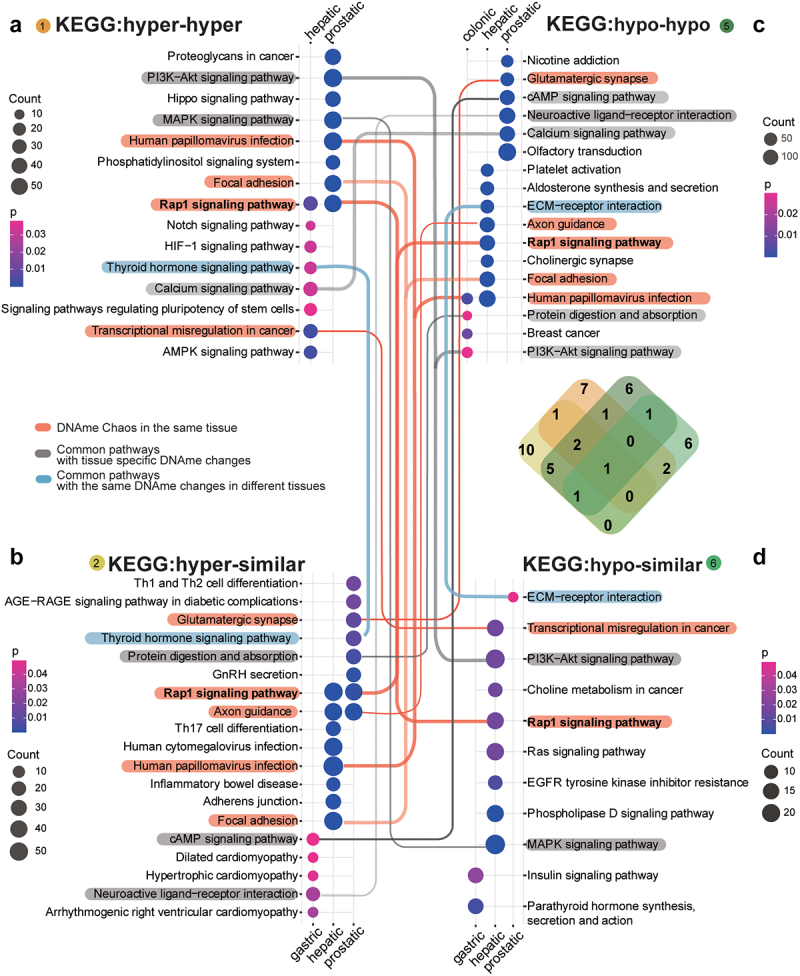
KEGG enrichment for methylation changes across the lesion progression stages.sHyperMethyl includes Types 1 (a) and 2 (b), and sHypomethyl contains Types 5 (c) and 6 (d). The lines indicate common enriched pathways. Red lines: pathways have both hyper and hypo-DNAme alterations enriched in the same tissue; grey lines: pathways harbour different types of DNAme changes in different tissues; blue lines: pathways have the same type of DNAme changes enriched in different tissues.

**Figure 4.**
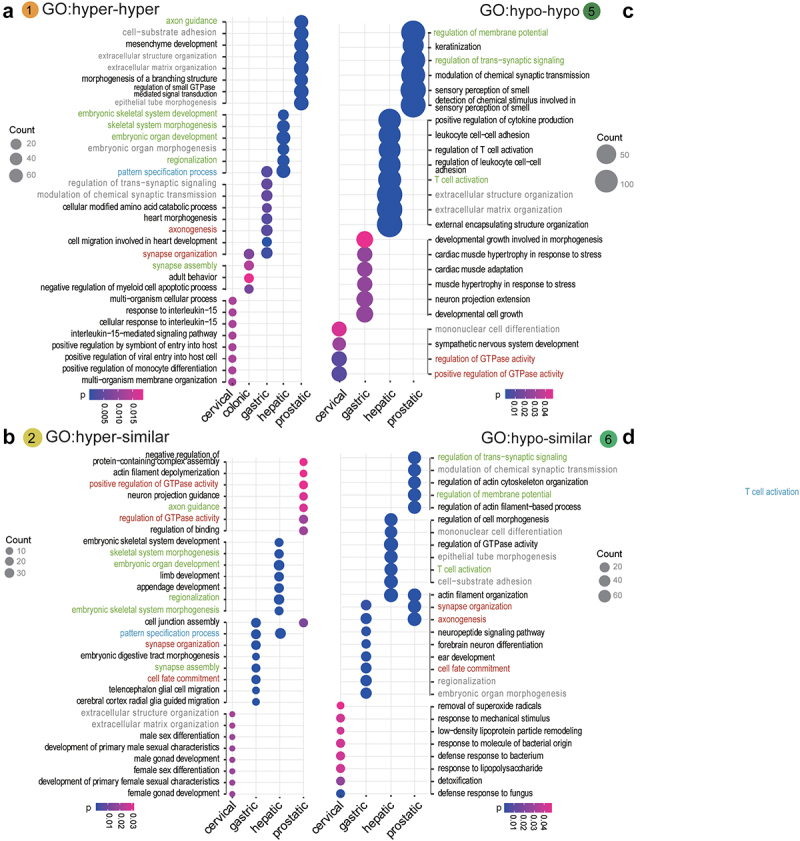
GO enrichment for methylation changes across the lesion progression stages. sHyperMethyl includes Types 1 (a) and 2 (b), and sHypomethyl contains Types 5 (c) and 6 (d). The pathways in red indicate common enriched pathways. Pathways in red have both hyper and hypo-DNAme alterations enriched in the same tissue; pathways in grey harbour different types of DNAme changes in different tissues; pathways in blue have the same type of DNAme changes enriched in different tissues; pathways in green have both types of either sHypermethyl or sHypomethyl enriched in the same tissue.

To characterize methylation changes across the lesion progression stages, we further analysed DMP-associated genes for hyper-hyper (Type 1), hyper-similar (Type 2), hypo-hypo (Type 5), and hypo-similar (Type 6) sites, among which sHyperMethyl indicated Types 1 and 2, and sHypoMethyl indicated Types 5 and 6. The common changes in the two stages may reflect the essential changes in tumorigenesis. The top enriched items, both for KEGG (Figure 3) and GO (Figure 4), showed obvious tissue specificity with few commonly affected functional processes (*P* value < 0.05, Pearson test). However, enrichment of the altered DNAme in the same KEGG pathways or GO items was still observed in multiple tissue lesions. Because of the large sample size, the number of differentially methylated sites was comparable for both prostatic and hepatic tissues. Thus, both common and distinct DNAme reprogramming processes indicating common biological processes in tumorigenesis of different tissues was demonstrated here. KEGG pathway analysis showed that the DNAme changes in both prostatic and hepatic lesions were enriched in the Rap1 signalling pathway. In prostatic lesions, only sHyperMethyl alterations were enriched in this pathway. However, in the progression from hepatic precancerous lesions to tumours, the Rap1 signalling pathway showed significant disorganization of methylation status, with both enrichment of sHyperMethyl and sHypoMethyl alterations, which were named as DNAme bidirectional chaos (Red lines, Figure 4). In hepatic lesions, DNAme bidirectional chaos was also enriched in ‘Human papillomavirus infection,’ ‘Focal adhesion,’ ‘Axon guidance’ and ‘Transcriptional misregulation in cancer.’ Prostatic lesions had even more DNAme changes than hepatic lesions, but the DNAme bidirectional chaos was not common, and was only enriched in ‘Glutamatergic synapse.’ Although DNAme changes were also enriched in ‘Human papillomavirus infection,’ ‘Focal adhesion,’ and ‘Axon guidance’ in the prostate dataset, they were all sHyperMethyl.

The accumulation of DNAme alterations demonstrated tissue specificity for affected pathways in different tissues. The PI3K−Akt signalling pathway showed sHyperMethyl enrichment in the prostate dataset, but sHypoMethyl enrichment was observed in the colonic and hepatic lesions. The same pattern was observed for the MAPK signalling pathway. Interestingly, GO enrichment results showed an even more pronounced tissue specificity than KEGG pathway enrichment (Figure 3 and 4). The identical alterations in the same pathway for different tissue lesions were relatively few. Most of the GO terms enriched for methylation changes in different tissue lesions exhibited different DNAme alterations (Grey front, Figure 4).

### Functions of loci with clinical survival prediction ability in eight types show significant tissue specificity

**Figure 5.**
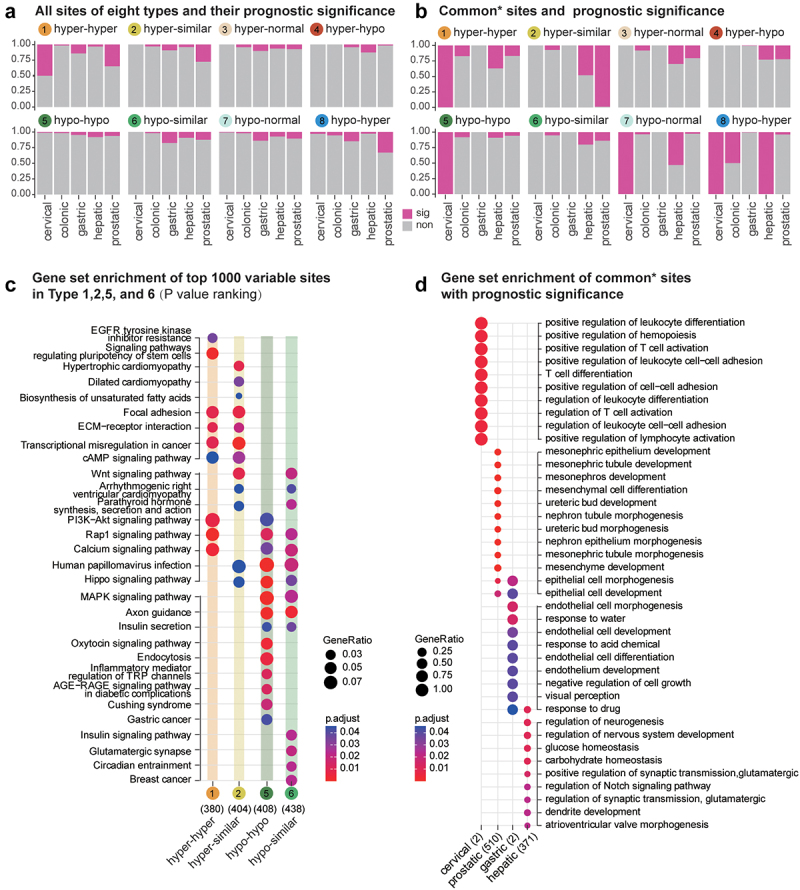
Survival prediction of DMPs across the precancerous state to the tumour.(a).Percentages of sites that had survival prediction ability in eight types of DMPs.(b).Percentages of sites that had survival prediction ability among common DMPs observed in more than two tissue types. (c). KEGG enrichment for the top 1000 sites demonstrates the most significant methylation alterations among different stages. (d). GO enrichment for common sites with survival prediction significance.

Next, we analysed whether these methylation changes, which were persistently retained in the diseased tissue, can affect the survival time of patients (Figure 5). Less than 25% of loci from each type (Figure 5a) were associated with patient survival. No type exhibited a significantly greater proportion of loci associated with patient survival. We further analysed the DNAme loci found in at least two tissues (common sites, Figure 5b) and did not find an elevated proportion of loci associated with patient survival. Thus, these altered loci (**Supplementary Table S1**), in most cases, did not lead to a more malignant phenotype of tumour cells. Considering the apparent tissue specificity shown in the functional enrichment analysis, these altered loci may simply reflect the required processes (sHyperMethyl and sHypoMethyl) or random events (other four types) that occur accidentally during the progression to the tumour.

Then, we ranked all the survival-associated loci according to their significance level between precancerous lesions and normal samples and analysed the common KEGG pathways in which the top 1000 sites may be involved (Figure 5c). In addition to the aforementioned pathways with DNAme bidirectional chaos in the hepatic dataset, the Wnt signalling pathway and Hippo signalling pathways were also affected by both sHyperMethyl and sHypoMethyl. For some other pathways, however, these most significant DNAme changes were manifested only as enrichment of either sHyperMethyl or sHypoMethyl. For instance, ‘EGFR tyrosine kinase inhibitor resistance’ (*P* = 0.0379, Type 1: hyper-hyper), ‘Signalling pathways regulating pluripotency of stem cells’ (*P* = 0.0014, Type 1: hyper-hyper), ‘ECM−receptor interaction’ (*P* = 0.0099, Type 1: hyper-hyper; *P* = 0.0219, Type 2: hyper-similar), ‘Transcriptional misregulation in cancer’ (*P* = 0.0104, Type 1: hyper-hyper; *P* = 0.000851, Type 2: hyper-similar), and ‘cAMP signalling pathway’ (*P* = 0.0485, Type 1: hyper-hyper; *P* = 0.0291, Type 2: hyper-similar) were featured by enrichment of sHyperMethyls; whereas, ‘MAPK signalling pathway’ (*P* = 0.000885, Type 5: hypo-hypo; *P* = 0.0254, Type 6: hypo-similar), ‘Oxytocin signalling pathway’ (*P* = 0.00473, Type 5: hypo-hypo), ‘Inflammatory mediator regulation of TRP channels’ (*P* = 0.013, Type 5: hypo-hypo), ‘Endocytosis’ (*P* = 0.00668, Type 5: hypo-hypo), and ‘Glutamatergic synapse’ (*P* = 0.0224, Type 6: hypo-similar) were characterized by enrichment of sHypoMethyls.

Additionally, we analysed the GO features of survival-associated loci that could be observed in more than two tumours among the eight types (Figure 5d, Supplementary Figure S4). Nevertheless, their functional enrichment still showed obvious tissue specificity. Particularly, the functions associated with mesenchymal stem cells (‘mesonephric epithelium development,’ ‘mesenchyme development,’ ‘mesonephric tubule morphogenesis,’ ‘nephron epithelium morphogenesis,’ ‘ureteric bud morphogenesis,’ ‘nephron tubule morphogenesis,’ ‘ureteric bud development,’ ‘mesenchymal cell differentiation,’ ‘mesonephros development,’ and ‘mesonephric tubule development’) were highlighted in the survival of prostate cancer patients. While, surprisingly the neurogenesis-related functions (‘regulation of neurogenesis,’ ‘regulation of nervous system development,’ ‘regulation of synaptic transmission, glutamatergic,’ ‘positive regulation of synaptic transmission, glutamatergic,’ and, ‘regulation of nervous system development’) were prominent in the survival of liver cancer patients. Although the loci for cervical and gastric datasets were limited, the enriched GO items underscored the importance of immune function in the prognosis of patients with cervical cancer and the role of epithelial cell-related functions in the survival of patients with gastric cancer.

### Common sHypermethyl and sHypomethyl alterations in the gene body of Onco/TSGs

**Figure 6.**
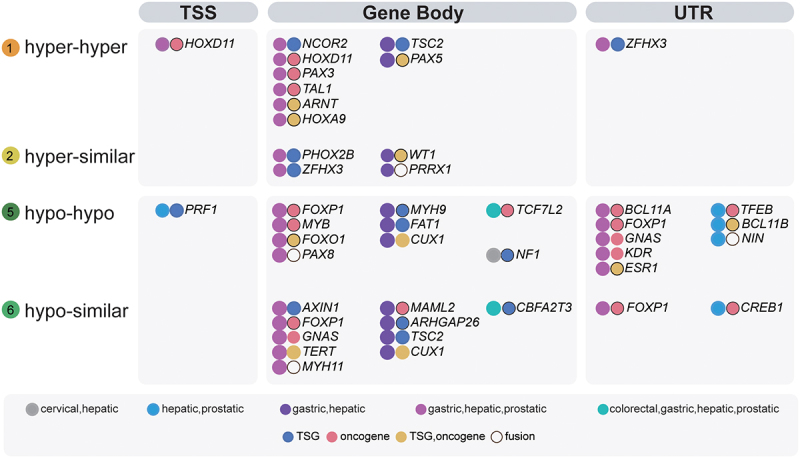
Distribution ofsHyperMethyl or sHypomethyl loci in gene models of TSGs and oncogenes.The three regions of TSS, Gene Body, and UTR were analysed.

For Onco/TSGs, we found 2852 sites in eight types of changes, which were located at 486 Onco/TSGs (**Supplementary Table S2**). Among them, there were 362 Onco/TSGs (362/486, 74%) harbouring the sHyperMethyl and sHypoMethyl changes (Types 1, 2, 5, 6 loci, 1244/2852, 41%). For the alterations observed in more than one tissue (Figure 6), 31 out of 36 Onco/TSGs harboured four types of DNAme alterations in their gene body, 11 had these alterations in the UTRs, and only 2 had these alterations in the TSS regions. Gastric, hepatic, and prostatic datasets shared the most Onco/TSG changes (Purple dots, Figure 6). The most common Onco/TSGs with altered methylation were *TCF7L2* and *CBFA2T3*, both carrying sHypoMethyl changes in colorectal, gastric, hepatic, and prostatic datasets. Among the corresponding tumours in the TCGA database, the overexpression of the oncogene *TCF7L2* was only found in gastric cancer (**Supplementary Figure S3A**). All these tumours had a slightly lower transcription of *CBFA2T3* (a TSG) (**Supplementary Figure S3B**).

In the three regions of UTR, gene body, and TSS, Onco/TSG DNAme alterations were most commonly observed in the gene body region, followed by the UTR, and then the TSS region. *FOXP1* showed the most methylation alterations among Onco/TSGs, with 4 sHypoMethyl alterations in its gene body (2/4) and UTR (2/4). The same gene could show methylation alterations in two regions simultaneously. The simultaneous occurrence of the methylation alterations in the gene body and UTR was more common than that in the gene body and TSS. The most common scenario was that both regions had the same methylation type. In the gene body and UTR regions, *FOXP1* and *GNAS* carried sHypoMethyl changes, while *ZFHX3* showed sHyperMethyl changes in gastric, hepatic, and prostatic datasets. In the gene body and TSS regions, *HOXD11* had sHyperMethyl alterations. For Onco/TSGs harbouring two DNAme alterations in the same region, in addition to the aforementioned *FOXP1, CUX1* also had two gene-body sHypoMethyls in both gastric and hepatic datasets. Nevertheless, in gastric and hepatic datasets, *TSC2* had one sHypoMethyl and one sHyperMethyl in the gene body.

There were gene families, which had no less than two members, affected by these DNAme alterations. *FOXO1* and *FOXP1* belong to the subfamily P of the forkhead box (FOX) transcription factor family. *HOXA9* and *HOXD11* are genes of the homeobox family, encoding a highly conserved family of transcription factors. *PAX3*, *PAX5*, and *PAX8* are members of the paired box (PAX) family of transcription factors. *MYH9* and *MYH11* come from the myosin heavy chain family. In addition, *BCL11A* and *BCL11B* are BAF Chromatin Remodeling Complex Subunits. They all had gene-body sHypoMethyl changes, except for *PAX3* and *PAX5*, both of which carried sHyperMethyls.

## Discussion

Precancerous lesions provide an opportunity to explore the avenue of tumorigenesis in different tissue. Herein, we analysed the similarities and differences of DNAme reprogramming during tumorigenesis in different tissues through the methylation profiles of precancerous lesions in different tumours. Our analysis demonstrated that the methylation levels showed a decreasing trend from normal to precancerous and tumour stages in all the analysed tissue samples except for cervical samples (Figure 1a). Consistently, recent studies have reported hypermethylation changes in cervical cancer. For example, El-Zein *et al*. found that the methylation levels significantly increased with lesion grade from normal to CIN1, CIN2, and CIN3 [37]. García *et al*. [38] identified 1069 hypermethylated promoter regions but only 85 hypomethylated regions in CIN III and cervical cancer in comparison with CIN I/II and normal tissues. Verlaat *et al*. [39] showed that the methylation levels of *GHSR*, *SST*, and *ZIC1* were increased with the increase of the cervical disease severity. In our study, the aDNAme level in cervical tissues (average β < 0.23) seemed to be significantly lower than that of other tissue types (average β, 0.41 ~ 0.49, Figure 1a). The extremely low aDNAme level in normal tissue may explain the seemingly hypermethylation in precancerous and cancer samples, and the whole aDNAme cannot become lower. Besides, global hypomethylation, as a well-known regional alteration of the tumour genome, is more common in the intergenic regions, which constitute the majority of the genome and cannot be fully covered by the probes of the microarray. Hence, whole genome sequencing is needed to cover the intergenic regions and to fully reveal the global methylation level in normal cervical tissue. Functional mechanisms underlying the low DNAme levels of normal cervical tissues deserve further in-depth analysis.

DNAme profiles have been widely used to cluster tumour samples into distinct groups with different phenotypes. In contrast, the aDNAme levels of normal tissues are believed to be consistent because of fewer genomic or epigenetic variations in normal tissues. The TCGA project enrols a smaller number of normal samples as controls. However, our analysis found this may not be the case in normal colorectal samples. They had the most significant aDNAme differences among samples in either TCGA or another public dataset (Figure 1a). There is also cell population heterogeneity in normal tissues. It has been highlighted that the extent of infiltrating immune cells varies across different normal tissues [40], and the immune cells, including T cells, Natural Killer (NK) cells, and B cells, are more abundant in colon tissue, than in the other four tissues analysed here. The individual differences in colorectal aDNAme level may be explained by the differences in cell population among samples. Although other tissues did not exhibit significant sample stratification under the current sample size, future epigenetic studies may require more normal controls to avoid potential stratification issues. Additionally, during the data pre-processing stage of integrating methylation profiles from different sources, we normalized the beta value of different tissues to avoid data overfitting, of which method was also been utilized in previous studies [41,42]. Moreover, in the following analysis, we prioritized identifying common changes observed in at least two tissues to minimize the impact of batch effects.

It is unlikely to obtain both precancerous and tumour samples from the same individual because the possibility of tumour occurrence is extremely low after the complete excision of precancerous lesions. Here, sHypoMethyls and sHyperMethyls were not inherited from precancerous lesions to tumours, but were common or similar alterations shared by both precancerous lesions and tumours. First, we hypothesized that some factors may be beneficial for the survival of tumour cells or may continue to promote DNAme changes during tumorigenesis, and grouped these changes into either similar levels in two stages (Type 2 for sHyperMethyls and Type 6 for sHypoMethyls) or more significant level changes in tumour (Type 1 for sHyperMethyls and Type 5 for sHypoMethyl). However, the definition of these loci was complicated by sample differences caused by cell population heterogeneity within each tissue bulk. So, we separated them for data visualization but combined them as the same type for interpretation. They were all crucial changes for both precancerous lesions and tumours, but the difference in the degree of their methylation levels between precancerous lesions and tumours needs to be verified by further studies with larger sample sizes. Different tissues perform their specific biological functions through active genes regulated by tissue-specific DNAme profiles and other factors, and tumours have common hallmarks [43]. Therefore, DNAme reprogramming from normal tissue to the tumour may demonstrate tissue specificity because the process initiates from tissue-specific DNAme profiles and evolves towards a relatively similar end. We showed that the tissue-specific KEGG pathways and GO terms were interrupted by DNAme alterations, and commonly affected pathways may be enriched by different types of methylation changes. Furthermore, DNAme changes related to the survival time of patients also demonstrated tissue specificity. The enriched genes included *CD4* in cervical cancer, *VPS52*, *NUP62*, *CRYGD*, *IL34*, *GATA3* in prostate cancer and *F11*, *TXNIP*, *KIT*, *ASGR2*, *SERPINF1* in liver cancer (**Supplementary Figure S4**), which also provide insight into the molecular mechanisms underlying cancer progression and the potential impact on patient survival.

Persistent DNAme alterations may be crucial for the early detection of tumours, understanding of tumorigenesis, and evaluation of tumour progression. Nevertheless, we did not find their increased performance in survival prediction, and the proportions of sites with prediction significance were not large for either sHyperMethyls or sHypoMethyls. This indicates that although DNAme alterations may be of importance in the formation of an epigenetic organization of the tumour genome, they may not directly lead to severe tumour phenotypes, the formation of which may need synergistic effects with other factors such as specific transcription factors [44]. In addition, genomic mutations may randomly interrupt these interactions during disease progression in different samples. Therefore, DNAme markers may be promising indicators for tumour screening, but may not necessarily affect disease prognosis alone. Additionally, epigenetic reprogramming of the tumour genome is complicated, as evidenced by DNAme bidirectional chaos in disrupted pathways, especially in tumorigenesis of liver cancer. Nevertheless, the common changes of Onco/TSGs in different tissues may highlight the key biological processes related to tumour occurrence in multiple tissues. Particularly, the hypomethylation changes in their gene bodies were most commonly observed. However, unlike the roles of gene-body hypermethylation in transcription regulation [45], the functions of gene-body hypomethylation still require extensive investigation. In addition, of all the 31 Onco/TSGs harboured sHyperMethyl and sHypoMethyl in their gene body, 7 genes (*HOXD11*, *TAL1*, *HOXA9*, *PAX5*, *FOXP1*, *FOXO1*, *TERT*) were reported to serve as potential DNA methylation-based biomarkers for non-invasive early cancer diagnosis [46–54]. Other Onco/TSGs genes have been proved potential functional role related to tumour development and the expression of some genes have been shown an emerging biomarker offering potential use in cancer diagnosis [55,56]. All these indicated that the sHyperMethyl and sHypoMethyl altered in the Onco/TSG gene body provided insights for early cancer diagnosis, which could be served as potential biomarkers with clinical application.

In summary, our findings in pan-precancerous DNAme alterations demonstrated the tissue-specific feature of epigenetic reprogramming in tumorigenesis, and highlighted the key interrupted processes in each tumour. The overall aDNAme level in cervical cancer seemed to be distinct from that of other tumours, characterized by an increased level of methylation in tumorigenesis, which may be related to the extremely low methylation levels in normal tissues. DNAme changes across the lesion progression stages illustrated the tissue specificity and epigenetic modification chaos of interrupted pathways, particularly in liver cancer. Gene-body hypomethylation should be paid more attention in future function investigation because it was a common alteration of Onco/TSGs across precancerous and cancer stages.

## Supplementary Material

Supplemental Material

## Data Availability

All data used in this study are available in the UCSC Xena website (https://xenabrowser.net/datapages/) and GEO (https://www.ncbi.nlm.nih.gov/geo/) with accession numbers of GSE13544, GSE135446, GSE68060, GSE139404, GSE99553, GSE103186, GSE115413, GSE157973. All the DMPs in five datasets are available at https://github.com/HaikunZhang1/PreCan.
