# Supplementary material for: Pan-precancer and cancer DNA methylation profiles revealed significant tissue specificity of interrupted biological processes in tumorigenesis
Source: Epigenetics. 2023 Jul 2;18(1):2231222. doi: 10.1080/15592294.2023.2231222 (PMC10316741; doi:10.1080/15592294.2023.2231222)
Supplement: Supplemental Material [file KEPI_A_2231222_SM3168.zip › Supplementary files/Supplementary Files captions.docx]

**Supplementary Files**

**Supplementary Figure 1** PCA analysis on samples from various tissues at three stages.

**Supplementary Figure 2** CpG isand distribution (A) and genomic features (B) of eight types of loci.

**Supplementary Figure 3** Gene transcription levels of *TCF7L2* (A) and *CBFA2T3* (B) in TCGA. Tumors in green show decreased transcriptional levels of the queried gene in comparison with normal tissue, and tumors in red had increased levels.

**Supplementary Figure 4** KM plots of survival-associated DMPs involved in the significant GO enrichment. Top 5 significant DMPs associated with OS in prostate cancer and liver cancer were displayed.

**Supplementary Table 1** DMPs show survival prediction ability.

**Supplementary Table 2** DMPs in TSG/Oncogene.
