# Supplementary figures and images for: Pan-precancer and cancer DNA methylation profiles revealed significant tissue specificity of interrupted biological processes in tumorigenesis
Source: Epigenetics. 2023 Jul 2;18(1):2231222. doi: 10.1080/15592294.2023.2231222 (PMC10316741; doi:10.1080/15592294.2023.2231222)

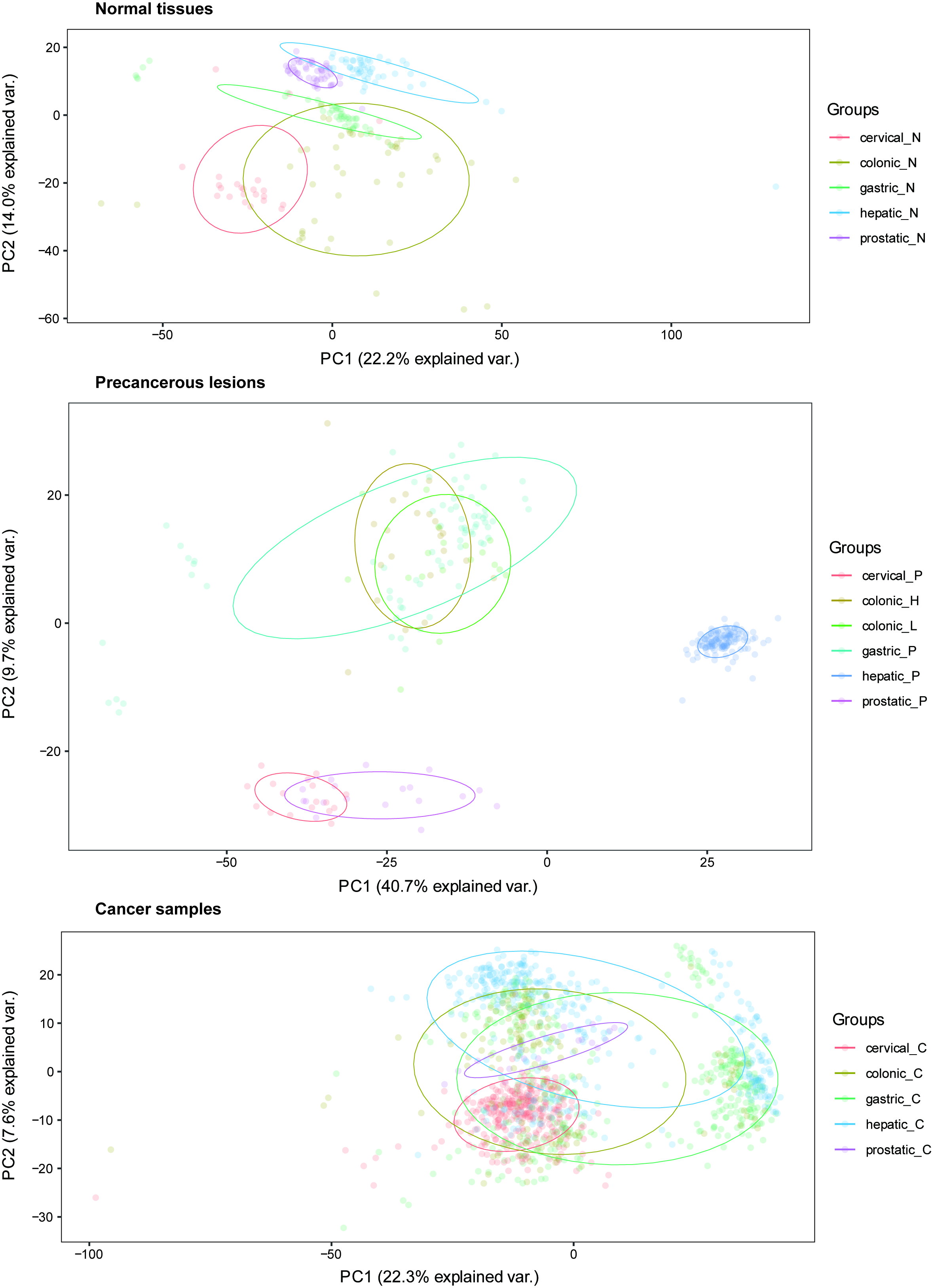

Supplement: Supplemental Material [file KEPI_A_2231222_SM3168.zip › Supplementary files/Supplementary Figure 1.tif]

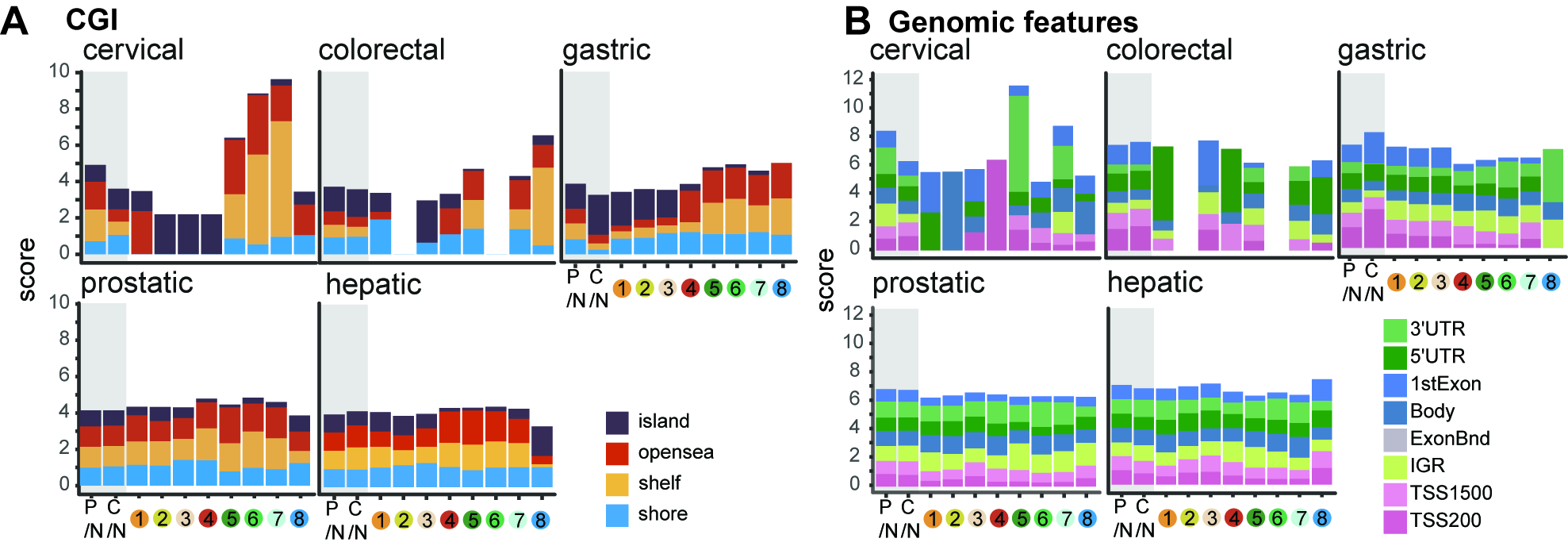

Supplement: Supplemental Material [file KEPI_A_2231222_SM3168.zip › Supplementary files/Supplementary Figure 2.tif]

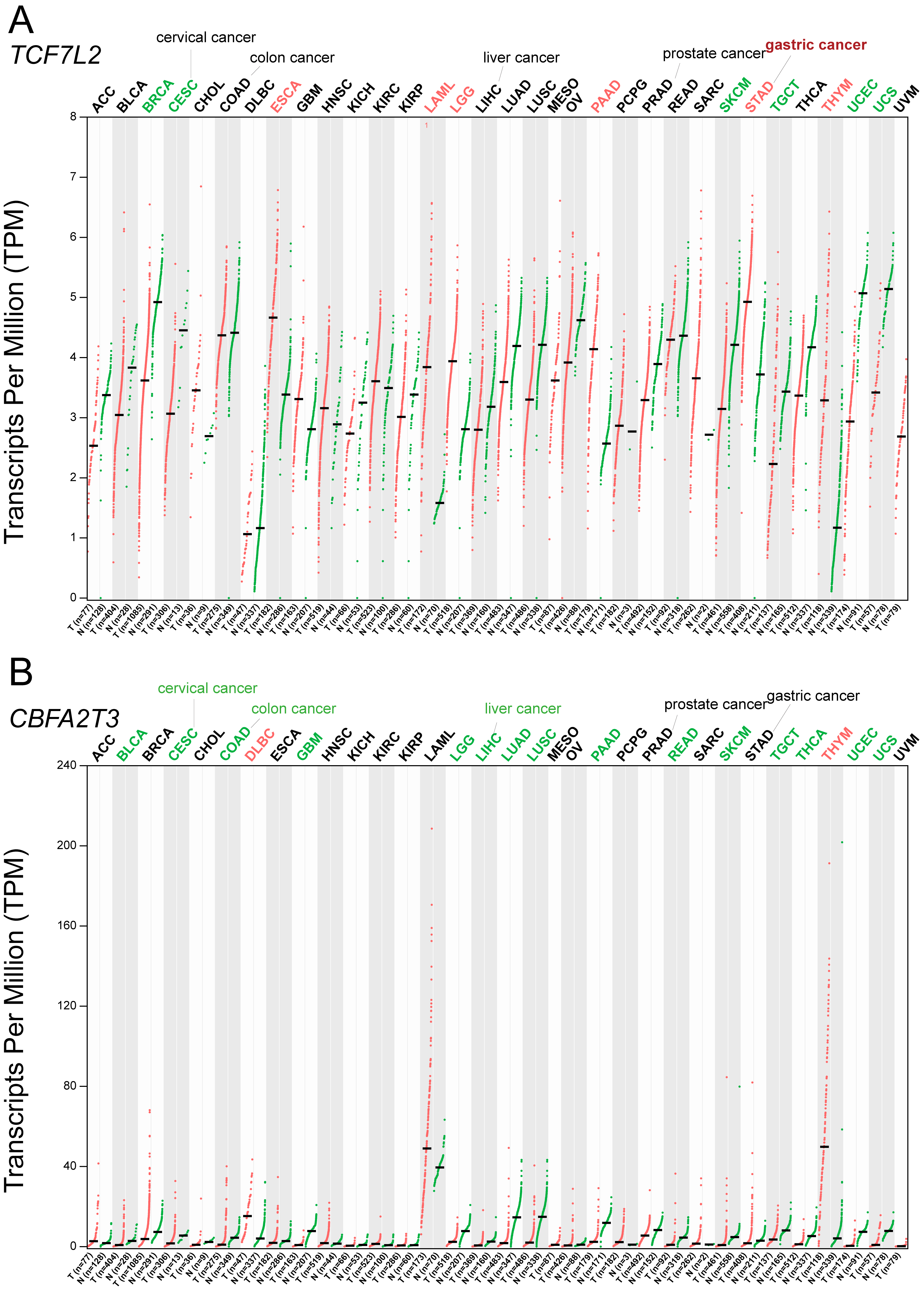

Supplement: Supplemental Material [file KEPI_A_2231222_SM3168.zip › Supplementary files/Supplementary Figure 3.tif]

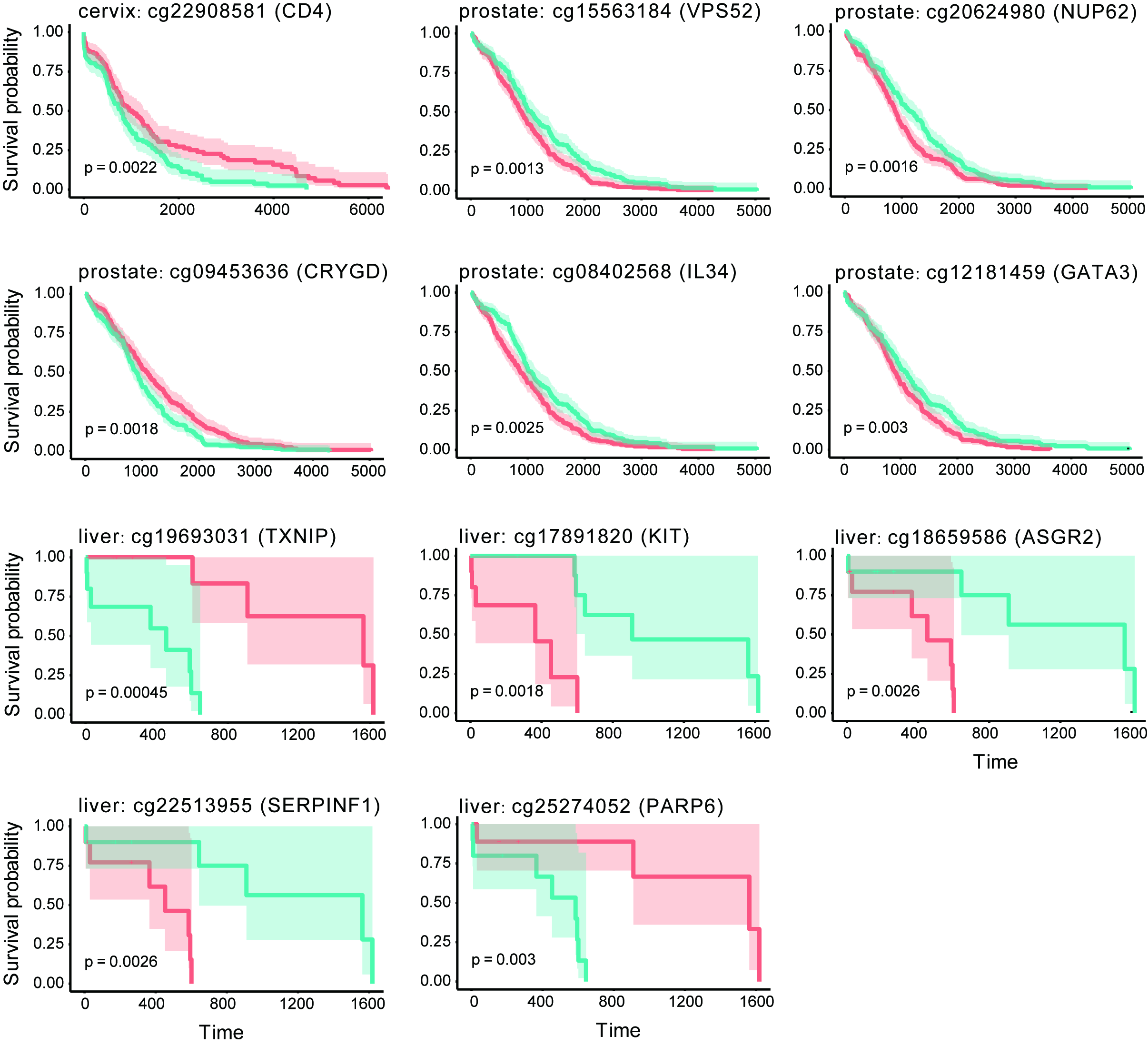

Supplement: Supplemental Material [file KEPI_A_2231222_SM3168.zip › Supplementary files/Supplementary Figure 4.tif]
